# Five novel mutations of the *FRMD7* gene in Chinese families with X-linked infantile nystagmus

**Published:** 2008-04-18

**Authors:** Ningdong Li, Liming Wang, Lihong Cui, Li Zhang, Suzhen Dai, Hongyan Li, Xia Chen, Lina Zhu, James F Hejtmancik, Kanxing Zhao

**Affiliations:** 1 Tianjin Eye Hospital, Tianjin Eye Institute, Tianjin, People’s Republic of China; 2 Tianjin Medical University, Tianjin, China; 3 HeNan Eye Institute, Zhengzhou, HeNan Province, China; 4 The Ophthalmic Department, the Central Hospital of Enshi Autonomous Prefecture, Hubei Province, China; 5 Ophthalmic Genetics and Visual Function Branch, National Eye Institute, National Institutes of Health, Bethesda, MD

## Abstract

**Purpose:**

Infantile nystagmus (IN) is an inherited disorder characterized by bilateral ocular oscillatory movements. Recently, mutations in FRMD7 were found to be responsible for X-linked idiopathic infantile nystagmus . We investigated the role of the FRMD7 gene mutations in seven Chinese families with infantile nystagmus.

**Methods:**

Linkage analysis was performed with fluorescently labeled microsatellite markers, DXS1001 and DXS1047. Analysis of *FRMD7* gene mutations was performed by direct sequence to the whole coding regions and exon-intron boundaries of *FRMD7* gene in all affected members in seven families with IN.

**Results:**

Five novel *FRMD7* gene mutations, 70 G>T(p.G24W) in exon 2, c.689–690delAG (p.Ser232del) in exon8, c. 782G>A (p.R260Q) and c. 812G>T (p. C271F) in exon 9, and c. 910C>T (R303X) in exon 10, were identified in five of seven Chinese families with X-linked infantile nystagmus. But we didn’t detect the *FRMD7* gene mutation in one of seven families, although a positive LOD score of 2.42 (θmax=0.1) was obtained at DXS1047 . We also found the same mutation, which is c. 782G>A (p.R260Q), occurred in two different families.

**Conclusions:**

This is first report that five kinds of *FRMD7* gene mutation types occurred in Chinese families with IN, which further support that *FRMD7* gene mutations are the underlying pathogenesis of the molecular mechanism for infantile nystagmus.

## Introduction

Infantile nystagmus (IN) is a relatively common ocular motor disorder characterized by rapid to and fro oscillations of the eyes. It usually presents itself at birth or develops within the first few months of life. The etiology of infantile nystagmus is not clear and usually occurs without sensory defect. This is different from “sensory defect nystagmus,” which is caused by inherited ocular diseases including albinism, achromatopsia, Leber congenital amaurosis, congenital cataract, and anterior segment dysgenesis.

Between 7% and 30% of IN patients have a positive family history [1] in which inheritance may be autosomal dominant, autosomal recessive, or X-linked. Five congenital nystagmus loci have been mapped including autosomal dominant NYS2 on chromosome 6p12 [2] (OMIM 164100), autosomal dominant NYS3 on chromosome 7p11.2 (OMIM 608345) [3], autosomal dominant NYS4 on chromosome 13q31-q22 (OMIM 193003) [4], and X-linked NYS1 on chromosome Xp11.4-p11.3 [5] (OMIM 310700) and Xq26-q27 [6]. X-linked inheritance is suggested to be the most common mode with incomplete penetrance in females [7].

Recently, mutations in *FRMD7* were found to be responsible for X-linked infantile nystagmus [8-10, 11,]. In this study, we report five novel mutations in *FRMD7* in Chinese families with infantile idiopathic nystagmus.

**Figure 1:**
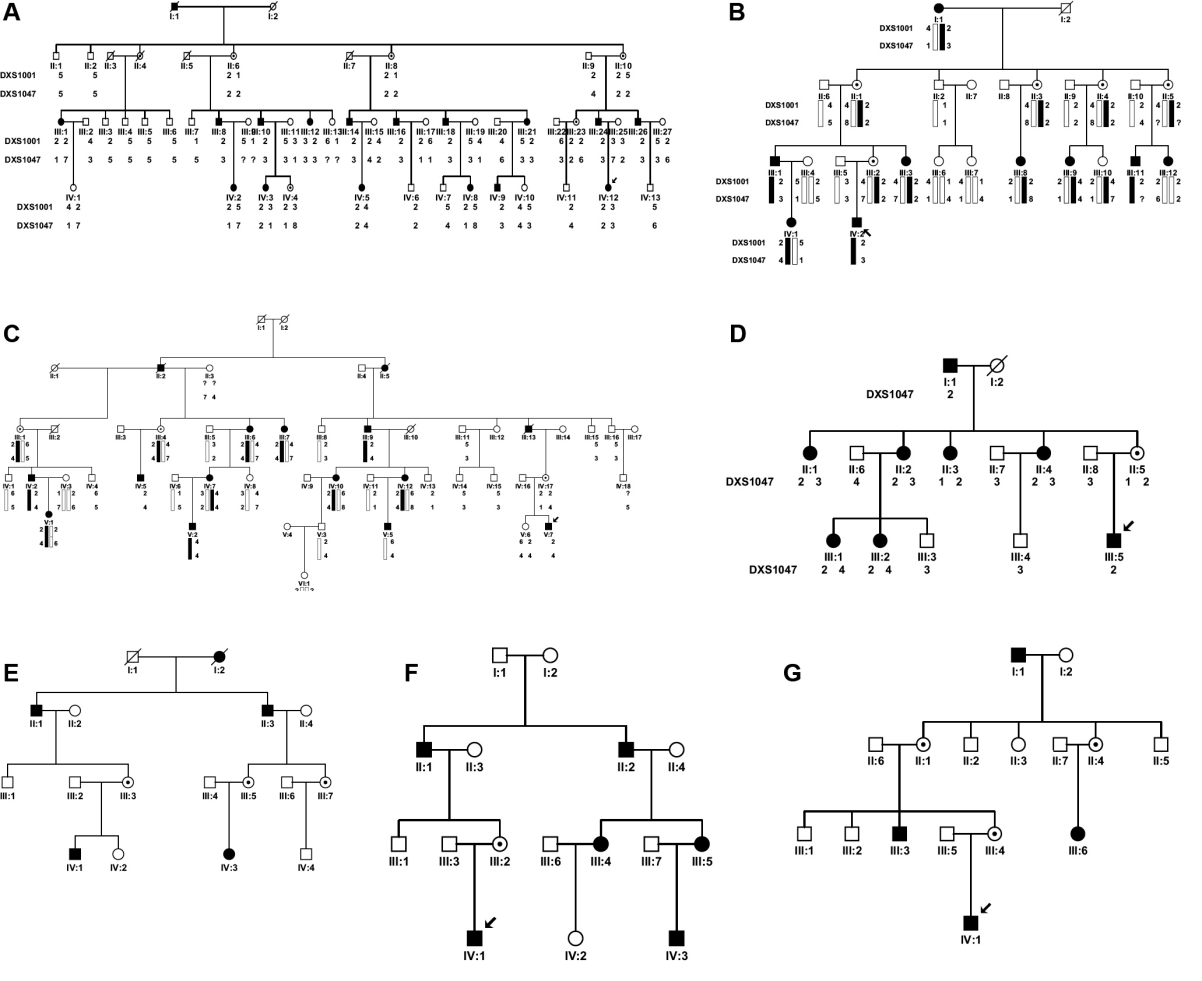
Pedigrees of seven families with X-linked congenital nystagmus. Squares indicate males, circles indicate females, slashed symbols indicate deceased, black symbols indicate affected individuals, unfilled symbols indicate unaffected individuals, and unaffected, obligate carriers are represented by a dotted circle.

## Methods

This study was approved by the Ethics Committee of the Tianjin Eye Hospital, Tianjin, China and was conducted according to the Tenets of the Declaration of Helsinki. Seven families with X-linked infantile idiopathic nystagmus were investigated .To exclude ocular or neural visual pathway abnormalities, a detailed clinical examination including visual acuities, slit examination of the lens, examination of the vitreous, fundus, electroretinogram (ERG), and visual evoked potential (VEP)was performed for those patients who could accept clinical examination at Tianjin Eye Hospital.

After informed consent was obtained, a 5 ml blood sample was collected from every participant and genomic DNA was isolated from peripheral blood lymphocytes using a DNA isolation kit (Roche Biochemical Inc., Basel, Switzerland.).

Linkage analysis was performed on those pedigrees with a sufficient number of affected individuals. Fluorescently labeled microsatellite markers, DXS1001 and DXS1047, were analyzed by polymerase chain reaction (PCR) amplification in a 10 μl reaction mixture containing 50 ng of genomic DNA, 1X PCR buffer, 2.0 mM MgCl_2_, 0.2 mmol/l of each dNTP, 5 pmol/l of each forward and reverse primer, and 0.2 U of AmpliTaq Gold DNA polymerase. Markers were genotyped using an ABI 3130 Genetic Analyzer (Applied Biosystems, Foster City, CA). Genotypes were analyzed using the GeneMapper 3.7 Software (Applied Biosystems).

Two-point LOD scores were calculated with easy Linkage plus v4.0 beta software, assuming an X-linked dominant trait with 100% penetrance on male and 50% in female, a disease allele of 0.001, and arbitrary marker allele frequencies of 1/n with n being the number of alleles observed for that specific marker.

Mutation screening was performed by direct DNA sequence analysis. Whole coding regions and exon-intron boundaries of the *FRMD7* gene were PCR-amplified in 50 μl of a standard PCR buffer containing 1.5 mmol/l MgCl_2_, 0.2 mmol/l of each dNTP, 0.5 μmol/l of each primer, 1 U of Taq polymerase, and 50 ng of DNA. The amplification program was an initial 2 min of denaturation at 98 °C followed by 30 cycles of 30 s at 94 °C, 30 s at 55 °C, 1 min at 72 °C, and a final 7 min extension step at 72 °C. The PCR products were extracted using the QIAquick Gel Extraction Kit (Qiagen, Valencia, CA). DNA sequencing analysis was performed using the BigDye Terminator Cycle Sequencing v3.1 kit on an ABI PRISM 3130 Genetic Analyzer (Applied Biosystems). Sequences were aligned using Chromas software (Conor McCarthy, Griffith University, Brisbane, Queensland,Australia). The reference cDNA sequence was obtained from the GenBank database (NM_194277.1), and +1 corresponds to the A of the ATG translation initiation codon.

All four SNPs are in the region 60 kb upstream of the start of the *FRMD7* gene transcript.

**Table 1:** Sequence analysis of four SNPs in two affected males from two families.

**Haplotype**	**Family**	**SNP**			
		rs2180237	rs1569893	rs2748723	rs2748724
II1	E	T	G	T	A
IV1	F	C	C	A	C

## Results

Four large families with infantile nystagmus were analyzed by linkage analysis. In family A (from the Sandong province), 16 of the 46 living family members are affected including 10 males and six females. In addition, three females are obligate unaffected carriers. No male-to-male transmission was observed. The proband is a nine-year-old girl (Figure 1A, individual IV_12_) who had nystagmus since three to four months after birth. She has a horizontal jerk ocular oscillation and a face turn to left with the null point to the right. The corrected visual acuity of both eyes at distance is 1.0. Her father is 32 years old with nystagmus since he was three to four months old. He has a horizontal ocular oscillation with a torsional component and with little head posture and head shaking. His corrected visual acuities for both eyes are 0.5. Other patients have various reduced visual acuity ranging from 0.2 to 1.0. Five of them have head posture, and two of them have head oscillations. The penetrance appears to be complete in males and about 60% in the females.

**Figure 2:**
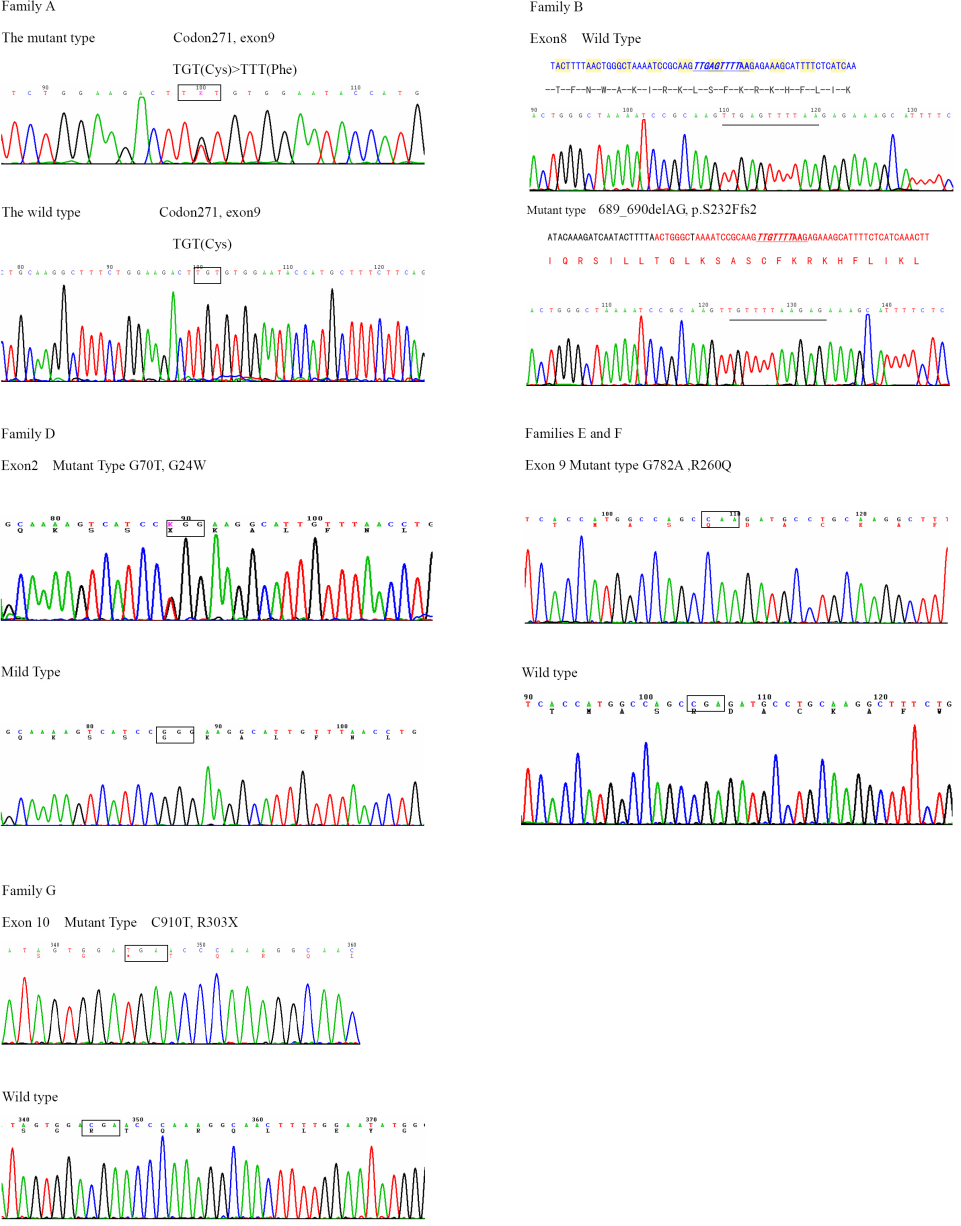
Sequencing results of affected heterozygous females from families A and D and of affected hemizygous males from families B, E, F, and G . Five types of *FRMD7* mutations were identified in six families, which were c. 812G>T (p. C271F) in family A, c.689–690delAG (p.Ser232del) in family B, c.70 G>T(p.G24W) in family D, c. 782G>A (p.R260Q) in both families E and F, and c. 910C>T (R303X) in family G.

Significant LOD scores were obtained with polymorphic markers DXS1047 (Z_max_=8.55, θ_max_=0) and DXS1001 (Z _max_=5.55, θ _max_=0). Direct DNA sequence analysis from all patients and obligate carriers reveals a c. 812G>T missense mutation in exon 9, which would result in a substitution of the cysteine residue at codon 271 by a phenylalanine residue (C271F, Figure 2A).

In family B (from the Henan province), nine patients (three males and six females) show horizontal jerk ocular oscillation. The affected males have more serious ocular oscillation than the affected females. Five females are obligate unaffected carriers. The proband is a nine-year-old boy (individual IV_2_ in Figure 1B) who developed nystagmus at three to four months of age with 0.7 corrected visual acuity in both eyes at distance. His mother is an asymptomatic obligate carrier. Other patients have various reduced visual acuity ranging from 0.4 to 1.0. A significant LOD Score of 3.61 (θ_max_=0) was obtained with polymorphic marker, DXS1047, and a LOD score of 2.03 (θ_max_=0.1) was obtained with DXS1001. Direct DNA sequence analysis of *FRMD7* from all patients and obligate carriers revealed a 2 bp deletion (AG, 689–690) in exon 8 that was not present in unaffected family members (Figure 2B). The deletion is predicted to result in an aberrant truncated protein.

Family C was from Liaoning province, and 12 patients (six males and six females) in this family were found with horizontal jerk ocular oscillations but without head posture. Three females are obligate unaffected carriers. The proband is an eight-year-old boy (individual V_7_ in Figure 1C) who developed nystagmus at three to four months of age with 0.3 corrected visual acuity of both eyes at distance. Other patients have various reduced visual acuity ranging from 0.2 to 1.0. Positive LOD scores of 2.42 (θ_max_=0.1) and 0.65 (θ_max_=0.25) were obtained with polymorphic markers, DXS1047 and DXS1001, respectively. No mutations were detected in the *FRMD7* gene, although all coding regions and exon-intron boundaries were sequenced.

In Family D (from Wuhan province), eight patients (two males and six females) show horizontal jerk ocular oscillation. The proband is a nine-year-old boy (individual III_5_ in Figure 1D), and his mother is the only asymptomatic obligate carrier in all the female members of this family. A positive LOD score of 1.57 (θ_max_=0) was obtained with polymorphic marker DXS1047, and a LOD score of 1.31 (θ_max_=0) was obtained with DXS1001. Sequence analysis revealed a c. 70G>T missense mutation in exon 2 in all patients and obligate carriers, which would result in a substitution of the glycine residue at codon 24 by a tryptophan residue (Figure 2D).

**Table 2:** Mutations in seven families and the significant LOD score in four families.

**Family**	**Location**	**Mutation**	**Protein**	**Type of mutation**	**Z_max_ at DXS1047**
A	exon 9	c. 812G>T	p.C271F	missense	8.55
B	exon 8	689–690delAG	p.S232Ffs2	deletion	3.61
C					2.42
D	exon 2	c. 70G>T	p.G24W	missense	1.57
E	exon 9	c. 782G>A	p.R260Q	missense	
F	exon 9	c. 782G>A	p.R260Q	missense	
G	exon 10	c. 910C>T	p.R303X	nonsense	

Families E and F are from Tianjin, and there is no indication by history that they are related. There is an insufficient number of available affected family members in these two families to provide a significant LOD score. A direct sequence of all affected patients in families E and F revealed the same sequence change in exon 9, a c. 782G>A missense mutation, which would result in substitution of the arginine residue at codon 260 by a glutamine residue (R260Q, Figure 2E,F).

To examine the possibility of a common origin between families E and F, four single nucleotide polymorphisms (SNPs; rs2180237, rs1569893, rs2748723, and rs2748724) in and around *FRMD7* were genotyped in affected males, individual II_1_ of family E and individual IV_1_ of family F (Table 1). The haplotypes of these two affected males and by extension that in their respective families is quite distinct. This suggests that the same mutation, R260Q, occurred independently in these two families rather than descending from a common ancestor.

Sequence analysis of all patients and obligate carriers in family G revealed a c. 910C>T nonsense mutation in exon 10, which would result in substitution of the arginine residue at codon 303 by a stop codon (R303X, Figure 2G).

The maximum LOD scores and mutations identified for each family are shown in Table 2. None of these five mutations were detected in 50 male and 50 female unaffected control individuals when tested by single strand conformation polymorphism (not shown). These results suggest that c.70 G>T (p.G24W) in exon 2, c.689–690delAG (p.Ser232del) in exon 8, c. 782G>A (p.R260Q) and c. 812G>T (p. C271F) in exon 9, and c. 910C>T (R303X) in exon 10 are novel mutations in the *FRMD7* gene.

## Discussion

In this study, we identified five novel mutations in the *FRMD7* gene. *FRMD7*, which has 12 exons and encodes 714 residues, is a previously unidentified member of the protein 4.1 superfamily. In situ hybridization experiments show that the *FRMD7* gene is expressed in the ventricular layer of the forebrain, midbrain, cerebellar primordium, spinal cord, and the developing neural retina in embryos 56 days post-ovulation. In earlier embryos 37 days post-ovulation, the expression is restricted to the midbrain and hindbrain, which are known to influence the motor control of eye movement [8].

**Figure 3:**
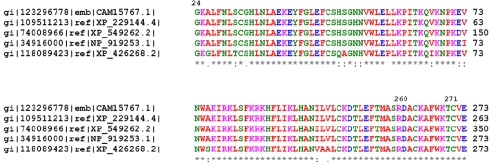
Alignment of FRMD7 amino acids. The alignment of amino acids around p.G24, p. R260, and p. C271 of FRMD7 revealed evolutionary conservation of the residue from *Canis familiaris*, *Rattus norvegicus*, *Mus musculus*, and *Gallus gallus* to *Homo sapiens*.

Previously, 32 mutations in the *FRMD7* gene have been identified in different ethnic groups [8-12] including G24R in exon 2 and C271Y in exon 9, which is similar to G24W and C271F identified in families D and A, respectively. We identified five novel mutations in all but one family investigated here, and family C in which no mutation was identified shows linkage to the *FRMD7* region. Missense, nonsense, and deletion mutations were observed. All changes we identified were novel mutations. The three missense mutations identified are G24W, C271F, and R260Q of which G24W and C271F occur at known mutation sites but substitute a different amino acid residue.

The FRMD7 protein is most highly conserved near the NH_2_-terminus, which includes the B41 and FERM-C domains. The B41 domain is located at residues 1–192, and the FERM-C domain is located at residues 186–279 in FRMD7. Modeling of the core domain of the cytoskeletal protein 4.1R suggests that mutations at codon 24 and codon 271 are likely to destabilize the protein by the introduction of larger amino acids within restricted areas of the protein [8]. Multiple alignment of the region shows that codon 24, codon 260, and codon 271 are located within highly conserved regions that are invariant in *Canis familiaris*, *Rattus norvegicus*, *Mus musculus*, and *Gallus gallus*, suggesting that these amino acids are critical to the normal function of the protein (Figure 3).

We also found a nonsense mutation, R303X, in exon 10 and a novel deletion mutation, p.S232Ffs2, in exon 8. Either of these two changes alters the length of the open reading frame, creating an aberrant truncated protein.

In one of our familial cases, no mutation was found in the coding region and exon-intron boundaries of *FRMD7*, although *FRMD7* was sequenced in all affected males and females. It is possible that congenital nystagmus in this family is caused by a mutation located in the promoter or other regulatory regions including those within introns or that there might be unknown splice variants that we didn’t detect with our experiment technique.

As shown by the results above, we have further expanded the mutational spectrum of *FRMD7* and confirmed that mutations in *FRMD7* are a common cause for X-linked congenital idiopathic nystagmus, although no mutations have been detected in a small subset of families mapped on Xq26–27. Further biochemical studies of the mutations in *FRMD7* will yield insight into its molecular mechanism underlying the pathogenesis of congenital idiopathic nystagmus.

## References

[1] Abadi RV, Bjerre A (2002). Motor and sensory characteristics of infantile nystagmus.. Br J Ophthalmol.

[2] Kerrison JB, Arnould VJ, Barmada MM, Koenekoop RK, Schmeckpeper BJ, Maumenee IH (1996). A gene for autosomal dominant congenital nystagmus localizes to 6p12.. Genomics.

[3] Klein C, Vieregge P, Heide W (1998). ET, al: Exclusion of chromosome regions 6p12 and 15q11, but not chromosome region 7p11, in a German family with autosomal dominant congenital nystagmus.. Genomics.

[4] Ragge NK, Hartley C, Dearlove AM, Walker J, Russell-Eggitt I, Harris CM (2003). Familial vestibulocerebellar disorder maps to chromosome 13q31-q33: a new nystagmus locus.. J Med Genet.

[5] Cabot A, Rozet JM, Gerber S (1999). etal: A gene for X-linked idiopathic congenital nystagmus (NYS1) maps to chromosome Xp11.4-p11.3.. Am J Hum Genet.

[6] Kerrison JB, Vagefi MR, Barmada MM, Maumenee IH (1999). Congenital motor nystagmus linked to Xq26–27.. Am J Hum Genet.

[7] Oetting WS, Armstrong CM, Holleschau AM, DeWan AT, Summers GC (2000). Evidence for genetic heterogeneity in families with congenital motor nystagmus (CN).. Ophthalmic Genet.

[8] Tarpey P, Thomas S, Sarvananthan N, Mallya U, Lisgo S, Talbot CJ, Roberts EO, Awan M, Surendran M, McLean RJ, Reinecke RD, Langmann A, Lindner S, Koch M, Jain S, Woodruff G, Gale RP, Degg C, Droutsas K, Asproudis I, Zubcov AA, Pieh C, Veal CD, Machado RD, Backhouse OC, Baumber L, Constantinescu CS, Brodsky MC, Hunter DG, Hertle RW, Read RJ, Edkins S, O’Meara S, Parker A, Stevens C, Teague J, Wooster R, Futreal PA, Trembath RC, Stratton MR, Raymond FL, Gottlob I (2006). Mutations in FRMD7, a newly identified member of the FERM family, cause X-linked idiopathic congenital nystagmus.. Nat Genet.

[9] Schorderet DF, Tiab L, Gaillard MC, Lorenz B, Klainquti G, Kerrison JB, Traboulsi EI, Munier FL (2007). Novel mutations in FRMD7 in X-linked congenital nystagmus. Mutation in brief #963. Online.. Hum Mutat.

[10] Zhang Q, Xiao X, Li S, Guo X (2007). FRMD7 mutations in Chinese families with X-linked congenital motor nystagmus.. Mol Vis.

[11] Zhang B, Liu Z, Zhao G, Xie X, Yin X, Hu Z, Xu S, Li Q, Song F, Tian J, Luo W, Ding M, Yin J, Xia K, Xia J (2007). Novel mutations of the FRMD7 gene in X-linked congenital motor nystagmus.. Mol Vis.

[12] Kaplan Y, Vargel I, Kansu T, Akin B, Rohmann E, Kamaci S, Uz E, Ozcelik T, Wollnik B, Akarsu NA (2008). Skewed X-inactivation in an X-linked Nystagmus Family Resulted from a Novel, p.R229G, Missense Mutation in the FRMD7 Gene.. Br J Ophthalmol.

